# Causal relationships between height, screen time, physical activity, sleep and myopia: univariable and multivariable Mendelian randomization

**DOI:** 10.3389/fpubh.2024.1383449

**Published:** 2024-06-20

**Authors:** Xiaoyu Liu, Fangkun Zhao, Weichen Yuan, Jun Xu

**Affiliations:** ^1^The Third People’s Hospital of Dalian, Dalian Municipal Eye Hospital, Dalian Municipal Cancer Hospital, Liaoning Provincial Key Laboratory of Cornea and Ocular Surface Diseases, Liaoning Provincial Optometry Technology Engineering Research Center, Dalian, Liaoning, China; ^2^Department of Ophthalmology, The Fourth Affiliated Hospital of China Medical University, Shenyang, China

**Keywords:** Mendelian randomization, myopia, risk factors, height, screen time, physical activity, sleep

## Abstract

**Background:**

This study aims to investigate the independent causal relation between height, screen time, physical activity, sleep and myopia.

**Methods:**

Instrumental variables (IVs) for exposures and outcome were obtained from the largest publicly available genome-wide association studies (GWAS) databases. First, we performed a bidirectional univariate MR analysis using primarily the inverse variance weighted method (IVW) with height, screen time, physical activity and sleep as the exposure and myopia as the outcome to investigate the causal relationship between exposures and myopia. Sensitivity analysis was used to demonstrate its robustness. Then the multivariable MR (MVMR) and MR-based mediation approach was further used to estimate the mediating effect of potential confounders (education and time outdoors) on causality.

**Results:**

The results of univariate MR analysis showed that taller height (OR = 1.009, 95% CI = 1.005–1.012, *p* = 3.71 × 10^−7^), longer time on computer (OR = 1.048, 95% CI = 1.029–1.047, *p* = 3.87 × 10^−7^) and less moderate physical activity (OR = 0.976, 95% CI = 0.96–0.991 *p* = 2.37 × 10^−3^) had a total effect on the increased risk of developing myopia. Meanwhile our results did not have sufficient evidence to support the causal relationship between chronotype (*p* = 0.637), sleep duration (*p* = 0.952) and myopia. After adjusting for education, only taller height remains an independent risk factor for myopia. After adjusting for education, the causal relationship between height, screen and myopia still had statistical significance. A reverse causal relationship was not found in our study. Most of the sensitivity analyses showed consistent results with those of the IVW method.

**Conclusion:**

Our MR study revealed that genetically predicted taller height, longer time on computer, less moderate physical activity increased the risk of myopia. After full adjustment for confounders, only height remained independently associated with myopia. As a complement to observational studies, the results of our analysis provide strong evidence for the improvement of myopia risk factors and provide a theoretical basis for future measures to prevent and control myopia in adolescents.

## Introduction

Myopia has become a growing public health concern worldwide, especially in some East and Southeast Asian regions with increasing incidence in recent decades (1). It is estimated that more than half of the world’s population will be affected by myopia by 2050 (2). High myopia (over −6.0 D) can even lead to pathological complications, such as cataracts, open-angle glaucoma and retinal detachment (3). Data from Asian populations show that myopia increases rapidly in childhood, affecting 80–90% of high school students, of whom 10–20% are high myopia (4). Myopia is a multifactorial disease related to genetic and environmental factors (5). We currently have strong evidence for a causal link between education and time outdoors and myopia (6). However, evidence remains weak and inconsistent on whether some highly concerned modifiable risk factors, such as height (7–10), screen time (11, 12), physical activity (13–16) and sleep (17–21), affect the development of myopia. Therefore, it is very important to further investigate the risk factors for myopia in order to propose feasible measures that can reduce the incidence of myopia and slow down the progression of myopia. Because the results of observational studies are inevitably affected by potential confounding factors and reverse causality. Randomized controlled trials (RCT) are also difficult to conduct due to the limitations of human and financial resources and ethics. In recent years, the genetics of myopia has flourished, with genome-wide association studies (GWAS) in particular successfully identifying many common genetic variants associated with myopia and refractive error (22). Genetic variation accounts for at least 12%, and possibly as much as 30% or more, of the variance in mean spherical equivalent refraction in populations of European ancestry at present (6). Under the circumstances, a well-designed mediation analysis and Mendelian randomization analysis would be particularly useful.

Mendelian randomization (MR) analysis employs genetic variations to evaluate the causality between exposures and outcomes and has the advantage of minimizing bias due to confounding factors and reverse causality (23). The study design is similar to randomized controlled trial (RCT) since genes are transferred from parents to offspring randomly (24). In this study, we conducted a MR analysis to thoroughly investigate about the causal effects of some highly discussed, clinically instructive and changeable personal factors, including height, screen time, physical activity, sleep on myopia using univariate MR and multivariate MR methods. Furthermore, we used MR-based mediation analysis to investigate whether education and time outdoors mediate the effects of exposures.

## Materials and methods

### Study design and MR assumptions

**Figure 1:**
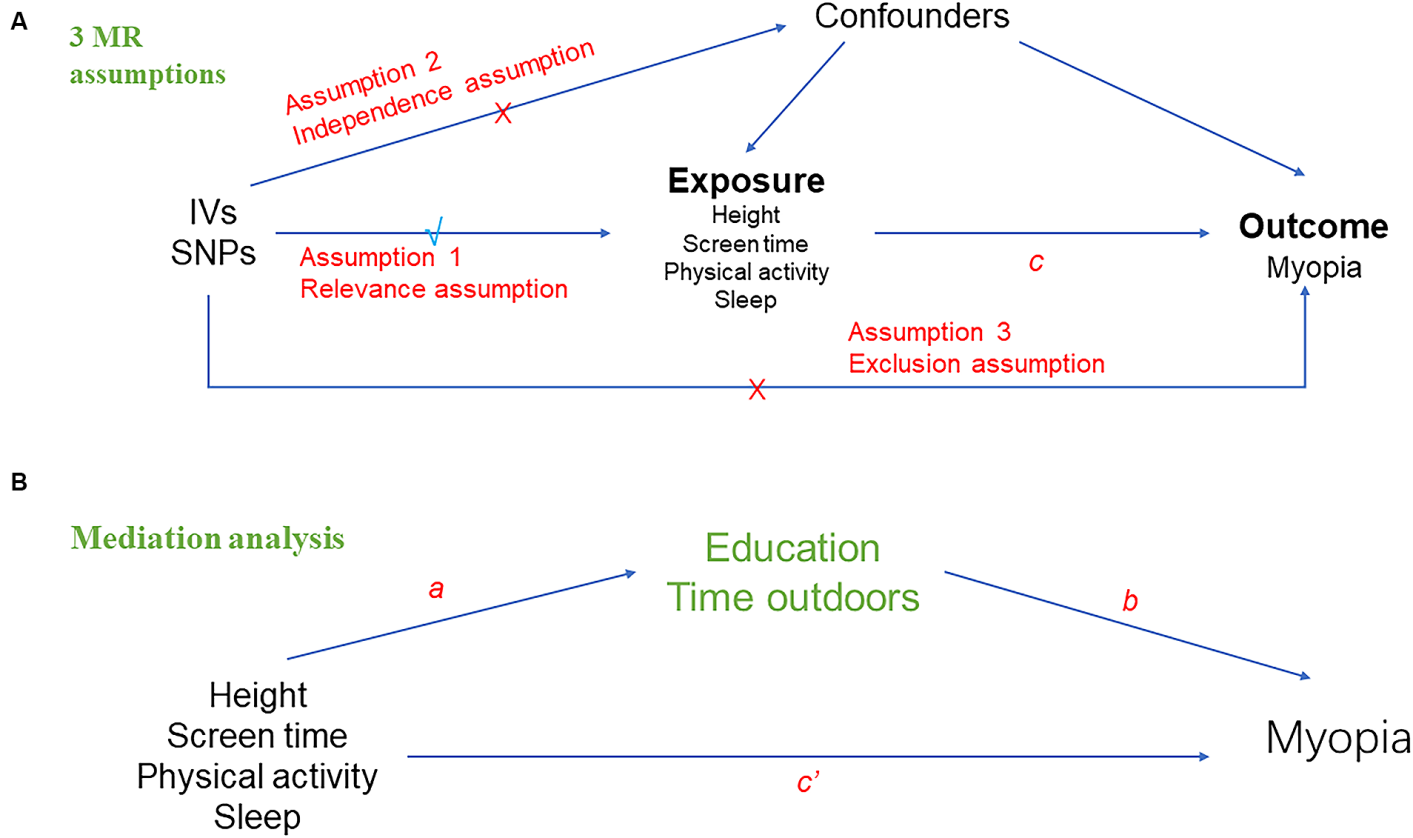
Overall design of Mendelian randomization analyses in the present study. **(A)** Schematic diagram of the three major assumptions of Mendelian randomization. **(B)** Mediation analysis diagram applied in this study.

In this study, we selected instrumental variables for the MR analysis using height, screen time, physical activity and sleep as ‘exposures’ and myopia as ‘outcome.’ Then, we conducted a two-sample bidirectional univariable MR analysis to evaluate the bidirectional causal relationships between exposures and myopia. Meanwhile sensitivity analysis was used to demonstrate its robustness. Next, we performed a multivariate MR analysis to assess whether the causal effect of exposures on myopia was mediated by education and time outdoors. Finally, a mediation analysis was calculated to assess the proportion of exposures’ effect on myopia mediated by education and time outdoors. Three assumptions must be satisfied in this MR analysis: (1) the genetic variant used in MR is associated with exposures (relevance assumption); (2) associations of the genetic variant with exposures and myopia must not be confounded (independence assumption); and (3) the genetic variants must have an association with myopia only through the effect associated with exposures (exclusion restriction assumption) (Figure 1A).

### Data source

The MR study was performed using publicly published studies or shared datasets. No additional ethics statement or consent was needed. Genetic instruments of height (standing height, *N* = 336,474), screen time (time spent using computer, *N* = 261,987) and physical activity (number of days/week of moderate physical activity 10+ minutes, *N* = 321,309) were derived from the Neale Lab consortium’s summary data from the UK Biobank. Genome-wide association study (GWAS) of chronotype (*N* = 413,343) and sleep duration (*N* = 460,099) extracted from the UK Biobank, a large cohort study with deep genetic and phenotypic data collected on more than 500,000 individuals from across the United Kingdom (25). Chronotype refers to an individual’s tendency for earlier or later sleep times, also known as circadian rhythm preference.

For the first assumption, the instrumental variables (IVs) for MR analyses were selected at a threshold of genome-wide significance (*p* < 5 × 10^−8^). Furthermore, the IVs for MR analyses were selected based on the following criteria: (1) To meet the MR assumptions, we performed a linkage disequilibrium (LD) analysis (*R*^2^ < 0.001, clumping distance = 10,000 kb) based on European-based 1,000 Genome Projects and removed the SNPs that did not meet the requirements. (2) Palindromic SNPs with intermediate allele frequencies were removed when harmonizing exposure and outcome data. (3) Proxy SNPs with *r*^2^ > 0.9 according to LDlink^1^ were used when the original SNPs were not available for the outcome. At the same time, we estimated the *F*-statistics to evaluate the instrument strength. *F* < 10 indicates weak instrument strength (26).

Myopia data integrated by the MRC IEU (“Phenotype: Reason for glasses/contact lenses: For short-sightedness, i.e., only or mainly for distance viewing such as driving, cinema etc., [called ‘myopia’]”) were used as an outcome, including 37,362 cases of myopia and 423,174 cases of population controls.

### MR analysis and sensitivity analysis

The principal method of two-sample MR conducted in this study was inverse variance weighted (IVW) (27), followed by MR-Egger and weighted median (WM) (28). If the assumption that all included SNPs can be used as valid IVs is met, the IVW method provides an accurate estimate (29). MR Egger’s results remained valid if SNPs with pleiotropy exceed 50%, but the estimation accuracy produced by this method is very low (30). WM analysis calculates the median of an empirical distribution of MR association estimates weighted for their accuracy, providing consistent estimates when more than half of the instruments are valid (28). Then, we performed reverse MR analysis with myopia as exposure. We adopted methods and settings that were consistent with the forward MR. Next, to investigate the direct effects of exposures on myopia, we performed multivariable MR (MVMR) analysis by multivariate random-effects IVW method, which is an extension of univariable MR that allows detecting causal effects of multiple risk factors jointly (31). Finally, we calculated the proportional effect of exposures on outcomes mediated by potential confounders (educations and time outdoors). A graphical summary of the analyses is given in Figure 1. First, we performed univariable two-sample MR to estimate the total effect of each exposure on myopia (c in Figure 1A) and the effect of each exposure on education and time outdoors (a in Figure 1B). We then used MVMR to estimate the effect of education and time outdoors on myopia (b in Figure 1B), adjusting for each exposure. Therefore, the indirect effect of each exposure on myopia, through education and time outdoors, was obtained by multiplying the effect of each exposure on each mediator and the effect of each mediator on myopia (a × b in Figure 1B). MR effect was considered significant at a Bonferroni-corrected *p* value of 0.05/5 = 0.01 (five exposure and one outcome). A *p* value between 0.01–0.05 was considered a suggestive association. The results were presented in odds ratios (OR) and 95% confidence intervals (CI).

Given that the traditional IVW MR method is susceptible to unbalanced horizontal pleiotropy (32). To assess comprehensive sensitivity, the heterogeneity estimated by Cochran’s *Q* test was used to appraise whether any single IV was driving the results and to check for consistency of the analyses with MR assumptions. The MR-Egger regression test intercept evaluates the evidence for directional pleiotropy, where intercepts that are significantly different from zero suggest directional pleiotropy (30) (*p* < 0.05). If the MR-PRESSO analysis indicated that significant outliers exist, we would remove the outlier variants and conduct the MR analysis again. Finally, we used leave-one-out plots for IVW estimates to confirm that the effects were not unduly influenced by outliers potentially representing pleiotropic pathways. All analyses are performed using the TwoSampleMR v0.5.7 package in the R software (Version 4.2.1), except for the MR-PRESSO model, which is performed using the MRPRESSO package v1.0.

## Results

### Univariable and bidirectional Mendelian randomization

**Figure 2:**
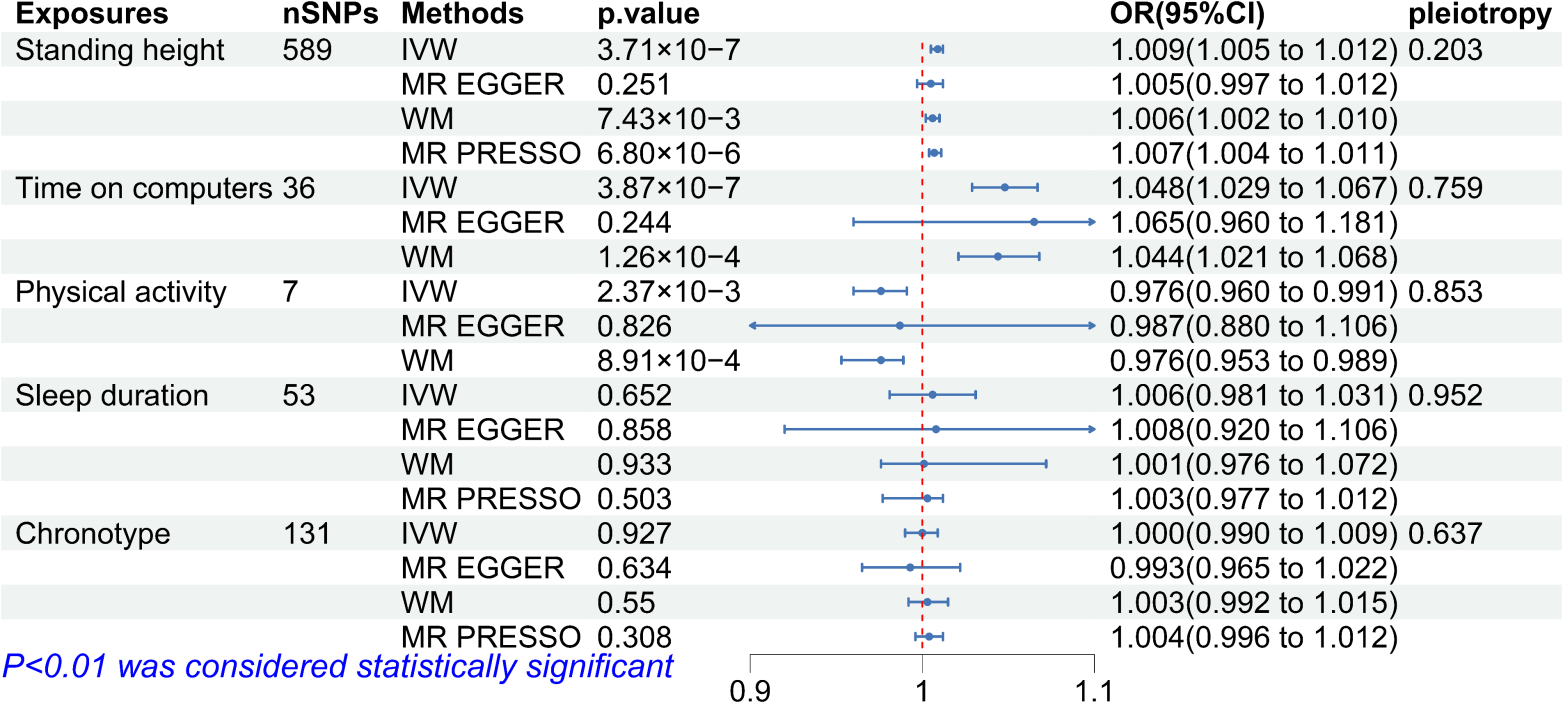
Mendelian randomization estimated effects of exposures on myopia.

After removing palindromic sequences, the number of SNPs included as IVs for exposures in MR analysis is shown in Figure 2. The detailed information for each SNP selected as IVs were shown in Supplementary Table 1. F-statistic for all exposures ranges from 15 to 667, indicating the absence of weak instrumental variables. The main IVW method shows that the taller height (OR = 1.009, 95%CI = 1.005–1.012, *p* = 3.71 × 10^−7^) and time spent on computer (OR = 1.048, 95%CI = 1.029–1.047, *p* = 3.87 × 10^−7^) may increase the risk of the onset of myopia and more physical activity is a protective factor against myopia (OR = 0.976, 95%CI = 0.96–0.991, *p* = 2.37 × 10^−3^). No significant causal relationships were found between sleep duration (OR = 1.006, 95%CI = 0.981–1.031, *p* = 0.652), chronotype (OR = 1, 95%CI = 0.99–1.009, *p* = 0.927) and myopia. More detailed results are shown in Figure 2. These associations are consistent across WM and MR-Egger methods, with similar causal estimates in direction and magnitude. Although the Cochran’s *Q* test on height, screen time, sleep duration and chronotype showed heterogeneity (*p* < 0.05), because we used random-effects IVW as the primary method, heterogeneity was acceptable and would not invalidate the estimate (33) (Supplementary Table 2). Meanwhile the causal relationship between exposures and myopia remained after excluding outliers using MR-PRESSO (Figure 2). The *p*-values of the intercepts were > 0.05 with the MR Egger pleiotropy test, indicating that there is no pleiotropic bias in the assessment of causal relationships with the IVW method. Leave-one-out analysis shows that all SNPs are evenly distributed on the same side of 0 and the causality is not driven by any single SNP. In the reverse MR analysis, we found no reverse causality between myopia and exposures.

### Multivariable Mendelian randomization and mediation analysis

Since the causal relationship between exposures and myopia might be partially mediated by education and time outdoors, we used MVMR analysis to assess the direct effect of exposure on myopia. According to the results of MVMR analysis, height can be considered an independent risk factor, because it still has a significant causal relationship with myopia after adjusting for education and outdoor time. Whereas physical activity does not appear to have a direct effect on myopia, as the significance disappears after adjustment. Meanwhile the causal relationship between screen time and myopia is largely mediated by education. Detailed data are shown in Table 1.

**Table 1:** The results of MVMR analysis and the proportion of mediation effects.

Exposure	Multivariate model	OR (95%CI)	*p* value	Mediation effect (%)
Height	MVMR adjusted for education	1.006 (1.003–1.01)	7.64 × 10^−8^*	31.4%
	MVMR adjusted for time outdoors	1.007 (1.004–1.011)	5.14 × 10^−5^*	18.8%
Time on computers	MVMR adjusted for education	1.005 (0.983–1.028)	0.648	64.5%
	MVMR adjusted for time outdoors	1.031 (1.012–1.05)	1.44 × 10^−3^*	46.5%
Physical activity	MVMR adjusted for education	0.993 (0.983–1.003)	0.185	15.8%
	MVMR adjusted for time outdoors	0.989 (0.974–1.004)	0.145	39.8%

*p < 0.01 is considered to be statistically significant.

Finally, we further calculated the proportional effect of height, screen time and physical activity on myopia mediated by education and time outdoors by mediation analysis. The results shown in Table 1 indicate that the effects of these exposures on myopia were all mediated to varying degrees by education and time spent outdoors.

## Discussion

Numerous observational studies have reported that height, screen time, physical activity and sleep may be risk factors for myopia, but these causal relationships have been inconsistent. Because it is difficult in observational studies to rule out the effects of known mediators and to avoid reverse causation. MR is a method of exploring the causal relationship between phenotypes and diseases at the genetic level using publicly available summary-level data, which overcomes the above shortcomings while simulating RCT. To our knowledge, we are the first to use MR to provide stronger evidence for the genetic association of these exposures with myopia. According to this MR study, taller height, longer time on computer and less moderate physical activity had a total effect on the increased risk of developing myopia. After adjusting for education, only taller height remains an independent risk factor for myopia. After adjusting for education, the causal relationship between height, screen and myopia still had statistical significance.

Many observational studies have reported a positive correlation between children’s height and axial length (AL) (7–10). In a cross-sectional observational study in Malaysia, Mohd-Ali et al. reported that for every centimeter increase in height, the longitudinal axial length of the eye increased by 0.056 mm (7). Tao et al. gave similar conclusions that there is a significant correlation between eye axis growth and height growth during the growth of preschool children in a 5-year cohort study (8). A recent longitudinal changes and cross-sectional analysis study came to the same conclusion in the post-COVID-19 era (34). Although it is well known that AL plays a crucial role in myopia, it does not mean that the lengthening of AL will necessarily increase the risk of the onset of myopia. In addition, Stickler syndrome is an autosomal recessive disease characterized by cleft palate, high myopia and increased height due to heterozygous pathogenic variants in the collagen genes COL2A1 and COL11A1, which to some extent suggests that there may be a potential genetic relationship between height and myopia (35). In contrary, some studies have suggested that adult height is independently related to ocular dimensions, but does not appear to have an effect on refraction (36). During the period of coordinated eye growth in children, the crystalline lens thins, which may also compensate for some myopia related to AL growth (37). In our MR study, similar to the results of most previous studies, we found that height had a genetic causal association with myopia (OR = 1.009, 95%CI = 1.005–1.012, *p* = 3.71 × 10^−7^). The results as shown in Figure 2 gave us sufficient confidence that height may increase the risk for short-sightedness. The statistical results are significant, although the OR value is not high. Some scholars believe that greater height and myopia are independent consequences of better socioeconomic status; therefore, there may be no causal relationship between height and myopia (38). At the same time, education, as an established risk factor for myopia (6), has an undeniable causal relationship with socioeconomic status. We therefore performed MVMR and mediation analysis to explore the direct causal relationship between height and myopia after adjusting for education. The results showed that the causal relationship between height and myopia remained after adjusting for education (OR = 1.006, 95%CI = 1.003–1.01, *p* = 7.64 × 10^−8^, 31.4%). Therefore, we can draw a conclusion that height has both total and direct genetic effect on myopia, with part of the effect mediated by education. The underlying biological mechanism for this causal relationship is unknown and some studies have reported that some systemic hormones may be regulators of longitudinal bone growth factors in childhood, which are also involved in the development of myopia (39).

Over the past decade, electronic devices such as computers and smartphones have taken up an increasing amount of children’s leisure and entertainment time with the rapid development of electronic information technology. It is reported that children are getting younger and younger to use smartphones (22% of children start at or under 3 years old), and 1/3 of children (1–6 years old) use smartphones for between 1 and 2 h a day (40, 41). Compared to traditional reading materials, smart devices can be watched for longer and closer distances, placing greater demand on accommodation and vergence (42). Therefore, whether screen use time, as a potential changeable risk factor, will increase the prevalence of myopia has become an urgent problem for parents and ophthalmologists. A recent systematic review and meta-analysis suggested that exposure to smart devices may be associated with an increase in the risk of myopia (11). Another systematic review involving 15 studies found insufficient evidence to show that there is a significant association between screen time and myopia prevalence, incidence or progression (12). Apparently, conclusions are inconsistent regarding the causal relationship between screen time and myopia. Long before the popularity of smart devices, myopia was widespread in East Asia. In recent years, as smartphones have become more integrated into every aspect of life, some recent observational studies have tended to confirm this link (12). However, it is difficult for observational studies to strictly control the mediating role of confounding factors such as education and outdoor activities in investigating the causal relationship between screen use time and myopia. According to the results of MR, we have reason to believe that the incidence of myopia will increase with the increase of time spent on computers (OR = 1.048, 95%CI = 1.029–1.047, *p* = 3.87 × 10^−7^). MVMR analysis results showed that after adjusting the outdoor time, the causality between them was still statistically significant (OR = 1.031, 95%CI = 1.012–1.05, *p* = 1.44 × 10^−3^, 46.5%). However, this causal relationship disappeared after adjusting for education (OR = 1.005, 95%CI = 0.983–1.028, *p* = 0.648), and 64.5% of the effect of computer use time on myopia was mediated by education. This result shown in Table 1 suggests that screen time has an overall effect on myopia, but it is largely mediated by education and has no direct effect. However, as screen time has continued to increase in recent years, this cannot be considered a final result. Recently, a large-scale intervention study from China showed that students’ online time was significantly positively associated with increased myopia incidence (43). Thus, the impact of screen time on myopia needs to be further evaluated.

Physical activity (PA) has a beneficial effect on the physical and cognitive health of school children, but the evidence is inconclusive as to whether it delays the onset and progression of myopia (44). Many well-designed observational studies have investigated the causal relationship between PA and myopia, but have not reached consistent conclusions (13–16). Liu et al. used a Bayesian model average to investigate the risk factors for myopia in adolescents, and the results showed that frequent participation in moderate sports activities was a powerful factor influencing the eyesight of middle school students (14). In contrast, seven-year longitudinal objective data on PA showed no significant association between PA and myopia during childhood (16). This discrepancy in results is not surprising since most studies do not exclude the regulation of outdoor time. However, what we want to investigate is whether indoor physical activity can be used as an intervention to prevent myopia, but this is difficult to design for observational studies. A previous detailed investigation concluded that among 12-year-old students, higher levels of outdoor activity were associated with lower prevalence of myopia while time spent in indoor sports had no effect (45). This conclusion is highly consistent with the results of our MR analysis. The MR analysis showed that the risk of myopia decreased with more frequent moderate physical activity of more than 10 min (OR = 0.976, 95%CI = 0.96–0.991, *p* = 2.37 × 10^−3^). However, this association became insignificant after adjusting for outdoor time (OR = 0.989, 95%CI = 0.974–1.004, *p* = 0.145) with a mediation effect of 39.8%. This means that even though physical activity has a total effect on the development of myopia, it is largely mediated by time outdoors and has no direct effect. A recent report provided a new perspective that physical activity may affect the onset and progression of myopia by improving children’s balance control (15). This means that increasing time outdoors may not be the only way for physical activity to reduce the risk of myopia, and further studies should be conducted to fully assess the complex relationship between them.

In recent years, many studies have been devoted to investigating the causal relationship between sleep and myopia, however the evidence provided is equivocal (17–21). Whether sleep duration and chronotype are modifiable risk factors for the development of myopia has also aroused the interest of many researchers. A recently published systematic review including a total of 31 studies with 205,907 participants showed that sufficient sleep duration was associated with a lower risk of myopia (46) (OR = 0.63, 95%CI = 0.51–0.78). In addition, a population-based cross-sectional study in Shanghai reported that not only was shorter sleep duration associated with myopia, but evening and intermediate chronotypes were positively correlated (47). The underlying mechanism for this causation may be related to the inactivity of the ciliary muscle, the self-regulation of the eye during sleep and the interaction of dopaminergic function with melatonin (48, 49). Evidence from animal studies also supports the notion that there are circadian or diurnal rhythms in parameters, such as axial length and choroidal thickness (48). Instead, the results of a cohort study design over a 4-year follow-up period showed that there was no significant association between sleep duration and myopia progression and axial elongation in primary school children (20). Consistently, according to our MR results, there is no sufficient evidence to suggest that there is a causal relationship between sleep duration, chronotype and myopia. The speculation about the difference in these consequences is that high educational intensity may make children spend more time on nearwork indoors, sacrificing the time spent exercising outdoor and sleeping, and mental activities before going to bed may also affect sleep duration and quality to some extent.

However, there are also several limitations in our MR research. First, the samples involved in our study are limited to the European population and may not apply to the Asian population with a higher incidence. Therefore, large-scale genetic data from more races are needed to help provide fully applicable conclusions on the causality. Second, as the outcome of this study, myopia is a binary variable and we can only explore whether there is a causal relationship between exposures and the onset of myopia but not the progression of myopia. Finally, we selected the GWAS data for exposures and outcome from publicly available summary data, however, it is impossible to determine whether overlapping subjects were included in our MR analysis.

In summary, this MR study revealed that genetically predicted taller height, longer time on computer and less moderate physical activity increased the risk of myopia. There is insufficient evidence for a genetic association between chronotype, sleep duration, and myopia. After full adjustment for known risk factors for myopia (education and time outdoors), only height remained independently associated with myopia. However, 64.5% of the effect of screen time on increased myopia risk was mediated by education. Physical activity does not appear to have a direct effect on the onset of myopia. As a complement to observational studies, the results of our analysis provide strong evidence for the improvement of myopia risk factors and provide a theoretical basis for future feasible measures to prevent and control myopia in adolescents.

## Data availability statement

The original contributions presented in the study are included in the article/Supplementary material, further inquiries can be directed to the corresponding author.

## Author contributions

XL: Data curation, Formal analysis, Investigation, Software, Visualization, Writing – original draft. FZ: Conceptualization, Funding acquisition, Methodology, Writing – original draft. WY: Conceptualization, Software, Writing – original draft. JX: Supervision, Writing – review & editing.

## Footnotes

^1^https://ldlink.nci.nih.gov/

## References

[1] Morgan IG French AN Ashby RS Guo X Ding X He M. The epidemics of myopia: aetiology and prevention. Prog Retin Eye Res. (2018) 62:134–49. doi: 10.1016/j.preteyeres.2017.09.004 28951126

[2] Holden BA Fricke TR Wilson DA Jong M Naidoo KS Sankaridurg P. Global prevalence of myopia and high myopia and temporal trends from 2000 through 2050. Ophthalmology. (2016) 123:1036–42. doi: 10.1016/j.ophtha.2016.01.006, PMID: 26875007

[3] Haarman AEG Enthoven CA Tideman JWL Tedja MS Verhoeven VJM Klaver CCW. The complications of myopia: a review and meta-analysis. Invest Ophthalmol Vis Sci. (2020) 61:49. doi: 10.1167/iovs.61.4.49, PMID: 32347918 PMC7401976

[4] Xu L Zhuang Y Zhang G Ma Y Yuan J Tu C. Design, methodology, and baseline of whole city-million scale children and adolescents myopia survey (CAMS) in Wenzhou, China. Eye Vis. (2021) 8:31. doi: 10.1186/s40662-021-00255-1, PMID: 34407890 PMC8373605

[5] Morgan IG Ohno-Matsui K Saw SM. Myopia. Lancet. (2012) 379:1739–48. doi: 10.1016/S0140-6736(12)60272-4 22559900

[6] Morgan IG Wu PC Ostrin LA Tideman JWL Yam JC Lan W. IMI risk factors for myopia. Invest Ophthalmol Vis Sci. (2021) 62:3. doi: 10.1167/iovs.62.5.3, PMID: 33909035 PMC8083079

[7] Mohd-Ali B Low YC Shahimin MM Arif N Abdul Hamid H Wan Abdul Halim WH. Ocular dimensions, refractive error, and body stature in Young Chinese children with myopia in Kuala Lumpur, Malaysia. Clin Optom. (2022) 14:101–10. doi: 10.2147/OPTO.S368672, PMID: 35910505 PMC9326897

[8] Tao L Wang C Peng Y Xu M Wan M Lou J. Correlation between increase of axial length and height growth in Chinese school-age children. Front Public Health. (2021) 9:817882. doi: 10.3389/fpubh.2021.817882 35127628 PMC8811027

[9] Yip VC Pan CW Lin XY Lee YS Gazzard G Wong TY. The relationship between growth spurts and myopia in Singapore children. Invest Ophthalmol Vis Sci. (2012) 53:7961–6. doi: 10.1167/iovs.12-10402, PMID: 23150611

[10] Wang D Ding X Liu B Zhang J He M. Longitudinal changes of axial length and height are associated and concomitant in children. Invest Ophthalmol Vis Sci. (2011) 52:7949–53. doi: 10.1167/iovs.11-7684, PMID: 21896861

[11] Foreman J Salim AT Praveen A Fonseka D Ting DSW Guang He M. Association between digital smart device use and myopia: a systematic review and meta-analysis. Lancet Digit Health. (2021) 3:e806–18. doi: 10.1016/S2589-7500(21)00135-7, PMID: 34625399

[12] Lanca C Saw SM. The association between digital screen time and myopia: a systematic review. Ophthalmic Physiol Opt. (2020) 40:216–29. doi: 10.1111/opo.12657, PMID: 31943280

[13] Hansen MH Laigaard PP Olsen EM Skovgaard AM Larsen M Kessel L. Low physical activity and higher use of screen devices are associated with myopia at the age of 16-17 years in the CCC2000 eye study. Acta Ophthalmol. (2020) 98:315–21. doi: 10.1111/aos.14242, PMID: 31502414

[14] Liu ZH Zhao MF Ma S Li Y Sun ZY Gao L. Exercise is the dominant factor affecting the development of teenagers' eyesight-based on the Bayesian model averaging. Front Public Health. (2022) 10:1014227. doi: 10.3389/fpubh.2022.1014227, PMID: 36589959 PMC9801519

[15] Modrzejewska M Domaradzki J Jedziniak W Florkiewicz B Zwierko T. Does physical activity moderate the relationship between myopia and functional status in children 9-11 years of age? J Clin Med. (2022) 11:5672. doi: 10.3390/jcm11195672, PMID: 36233536 PMC9572250

[16] Lundberg K Suhr Thykjaer A Sogaard Hansen R Vestergaard AH Jacobsen N Goldschmidt E. Physical activity and myopia in Danish children-the CHAMPS eye study. Acta Ophthalmol. (2018) 96:134–41. doi: 10.1111/aos.13513, PMID: 28671340

[17] Stone RA McGlinn AM Chakraborty R Lee DC Yang V Elmasri A. Altered ocular parameters from circadian clock gene disruptions. PLoS One. (2019) 14:e0217111. doi: 10.1371/journal.pone.0217111, PMID: 31211778 PMC6581257

[18] Jee D Morgan IG Kim EC. Inverse relationship between sleep duration and myopia. Acta Ophthalmol. (2016) 94:e204–10. doi: 10.1111/aos.12776, PMID: 26031352

[19] Pan CW Liu JH Wu RK Zhong H Li J. Disordered sleep and myopia among adolescents: a propensity score matching analysis. Ophthalmic Epidemiol. (2019) 26:155–60. doi: 10.1080/09286586.2018.1554159, PMID: 30601071

[20] Wei SF Li SM Liu L Li H Kang MT Sun YY. Sleep duration, bedtime, and myopia progression in a 4-year follow-up of Chinese children: the Anyang childhood eye study. Invest Ophthalmol Vis Sci. (2020) 61:37. doi: 10.1167/iovs.61.3.37, PMID: 32196099 PMC7401424

[21] Huang L Chen X Lin J Fan X Chen T Yu Y. Association between sleep duration and myopia among Chinese children during the COVID-19 pandemic: a cross-sectional study. Front Public Health. (2022) 10:1015138. doi: 10.3389/fpubh.2022.1015138 36699911 PMC9868807

[22] Tedja MS Haarman AEG Meester-Smoor MA Kaprio J Mackey DA Guggenheim JA. IMI-myopia genetics report. Invest Ophthalmol Vis Sci. (2019) 60:M89–M105. doi: 10.1167/iovs.18-25965, PMID: 30817828 PMC6892384

[23] Emdin CA Khera AV Kathiresan S. Mendelian randomization. JAMA. (2017) 318:1925–6. doi: 10.1001/jama.2017.17219 29164242

[24] Smith GD Ebrahim S. 'Mendelian randomization': can genetic epidemiology contribute to understanding environmental determinants of disease? Int J Epidemiol. (2003) 32:1–22. doi: 10.1093/ije/dyg070, PMID: 12689998

[25] Bycroft C Freeman C Petkova D Band G Elliott LT Sharp K. The UK biobank resource with deep phenotyping and genomic data. Nature. (2018) 562:203–9. doi: 10.1038/s41586-018-0579-z, PMID: 30305743 PMC6786975

[26] Burgess S Thompson SG Collaboration CCG. Avoiding bias from weak instruments in Mendelian randomization studies. Int J Epidemiol. (2011) 40:755–64. doi: 10.1093/ije/dyr036, PMID: 21414999

[27] International Consortium for Blood Pressure Genome-Wide Association Studies Ehret GB Munroe PB Rice KM Bochud M Johnson AD. Genetic variants in novel pathways influence blood pressure and cardiovascular disease risk. Nature. (2011) 478:103–9. doi: 10.1038/nature10405, PMID: 21909115 PMC3340926

[28] Bowden J Davey Smith G Haycock PC Burgess S. Consistent estimation in Mendelian randomization with some invalid instruments using a weighted median estimator. Genet Epidemiol. (2016) 40:304–14. doi: 10.1002/gepi.21965, PMID: 27061298 PMC4849733

[29] Burgess S Butterworth A Thompson SG. Mendelian randomization analysis with multiple genetic variants using summarized data. Genet Epidemiol. (2013) 37:658–65. doi: 10.1002/gepi.21758, PMID: 24114802 PMC4377079

[30] Bowden J Davey Smith G Burgess S. Mendelian randomization with invalid instruments: effect estimation and bias detection through egger regression. Int J Epidemiol. (2015) 44:512–25. doi: 10.1093/ije/dyv080, PMID: 26050253 PMC4469799

[31] Sanderson E Davey Smith G Windmeijer F Bowden J. An examination of multivariable Mendelian randomization in the single-sample and two-sample summary data settings. Int J Epidemiol. (2019) 48:713–27. doi: 10.1093/ije/dyy262, PMID: 30535378 PMC6734942

[32] Holmes MV Ala-Korpela M Smith GD. Mendelian randomization in cardiometabolic disease: challenges in evaluating causality. Nat Rev Cardiol. (2017) 14:577–90. doi: 10.1038/nrcardio.2017.78, PMID: 28569269 PMC5600813

[33] Burgess S Davey Smith G Davies NM Dudbridge F Gill D Glymour MM. Guidelines for performing Mendelian randomization investigations. Wellcome Open Res. (2019) 4:186. doi: 10.12688/wellcomeopenres.15555.1 32760811 PMC7384151

[34] Du W Ding G Guo X Abudukeyimu K Wang Y Wang L. Associations between anthropometric indicators and refraction in school-age children during the post-COVID-19 era. Front Public Health. (2022) 10:1059465. doi: 10.3389/fpubh.2022.1059465 36743176 PMC9891462

[35] Nixon TRW Richards AJ Martin H Alexander P Snead MP. Autosomal recessive stickler syndrome. Genes. (2022) 13:7. doi: 10.3390/genes13071135 PMC9324312 35885918

[36] Wong TY Foster PJ Johnson GJ Klein BE Seah SK. The relationship between ocular dimensions and refraction with adult stature: the Tanjong Pagar survey. Invest Ophthalmol Vis Sci. (2001) 42:1237–42. PMID: 11328733

[37] Zadnik K Mutti DO Fusaro RE Adams AJ. Longitudinal evidence of crystalline lens thinning in children. Invest Ophthalmol Vis Sci. (1995) 36:1581–7. PMID: 7601639

[38] Saw SM Chua WH Hong CY Wu HM Chia KS Stone RA. Height and its relationship to refraction and biometry parameters in Singapore Chinese children. Invest Ophthalmol Vis Sci. (2002) 43:1408–13. PMID: 11980854

[39] Rada JA Wiechmann AF. Ocular expression of avian thymic hormone: changes during the recovery from induced myopia. Mol Vis. (2009) 15:778–92. PMID: 19390653 PMC2671582

[40] Tan QYJ Hartanto A Toh WX Yang H. Commentary: influence of smartphone addiction proneness of young children on problematic behaviors and emotional intelligence: mediating self-assessment effects of parents using smartphones. Front Psychol. (2019) 10:115. doi: 10.3389/fpsyg.2019.00115, PMID: 30834900 PMC6387899

[41] Bernard JY Padmapriya N Chen B Cai S Tan KH Yap F. Predictors of screen viewing time in young Singaporean children: the GUSTO cohort. Int J Behav Nutr Phys Act. (2017) 14:112. doi: 10.1186/s12966-017-0562-3, PMID: 28870219 PMC5584344

[42] Bababekova Y Rosenfield M Hue JE Huang RR. Font size and viewing distance of handheld smart phones. Optom Vis Sci. (2011) 88:795–7. doi: 10.1097/OPX.0b013e3182198792, PMID: 21499163

[43] Xu L Ma Y Yuan J Zhang Y Wang H Zhang G. COVID-19 quarantine reveals that behavioral changes have an effect on myopia progression. Ophthalmology. (2021) 128:1652–4. doi: 10.1016/j.ophtha.2021.04.001, PMID: 33857574 PMC8463956

[44] Janssen I Leblanc AG. Systematic review of the health benefits of physical activity and fitness in school-aged children and youth. Int J Behav Nutr Phys Act. (2010) 7:40. doi: 10.1186/1479-5868-7-40, PMID: 20459784 PMC2885312

[45] Rose KA Morgan IG Ip J Kifley A Huynh S Smith W. Outdoor activity reduces the prevalence of myopia in children. Ophthalmology. (2008) 115:1279–85. doi: 10.1016/j.ophtha.2007.12.019 18294691

[46] Wang XX Liu X Lin Q Dong P Wei YB Liu JJ. Association between sleep duration, sleep quality, bedtime and myopia: a systematic review and meta-analysis. Clin Experiment Ophthalmol. (2023) 51:673–84. doi: 10.1111/ceo.14277, PMID: 37468126

[47] Li R Chen Y Zhao A Huang L Long Z Kang W. Relationships between sleep duration, timing, consistency, and Chronotype with myopia among school-aged children. J Ophthalmol. (2022) 2022:1–11. doi: 10.1155/2022/7071801 PMC9325560 35903175

[48] Nickla DL. Ocular diurnal rhythms and eye growth regulation: where we are 50 years after Lauber. Exp Eye Res. (2013) 114:25–34. doi: 10.1016/j.exer.2012.12.013, PMID: 23298452 PMC3742730

[49] Feldkaemper M Schaeffel F. An updated view on the role of dopamine in myopia. Exp Eye Res. (2013) 114:106–19. doi: 10.1016/j.exer.2013.02.007, PMID: 23434455

